# Reversible Valproate-Induced Subacute Encephalopathy Associated With a *MT-ATP8* Variant in the Mitochondrial Genome

**DOI:** 10.3389/fneur.2018.00728

**Published:** 2018-08-30

**Authors:** Giovanna De Michele, Pierpaolo Sorrentino, Claudia Nesti, Anna Rubegni, Lucia Ruggiero, Silvio Peluso, Antonella Antenora, Mario Quarantelli, Alessandro Filla, Giuseppe De Michele, Filippo M. Santorelli

**Affiliations:** ^1^Department of Neurosciences and Reproductive and Odontostomatological Sciences, Università degli Studi di Napoli Federico II, Napoli, Italy; ^2^Molecular Medicine, IRCCS Fondazione Stella Maris, Pisa, Italy; ^3^Institute of Biostructure and Bioimaging, National Research Council, Naples, Italy

**Keywords:** valproate, mitochondria, *MT-ATP8*, metabolic encephalopathy, ammonia

## Abstract

**Introduction:** There are several reported cases of patients developing motor and cognitive neurological impairment under treatment with valproic acid (VPA). We describe a woman who developed a subacute encephalopathy after VPA intake, harboring a mitochondrial DNA variant, previously described as causing VPA sensitivity in one pediatric patient.

**Material and Methods:** A 65-year old woman developed a progressive, severe neurological deterioration after a 3 month treatment with valproate sodium, 800 mg daily. Magnetic resonance spectroscopy (MRS), muscle histochemical analysis and assay of mitochondrial enzymatic activities, and mitochondrial DNA sequencing were performed.

**Results:** Neurological examination showed drowsiness, vertical gaze palsy, inability to either stand or walk, diffuse weakness, increased tendon reflexes. Blood lactate was increased, EEG showed diffuse theta and delta activity, MRI subcortical atrophy and leukoencephalopathy, MRS marked reduction of the NAA spectrum, with a small signal compatible with presence of lactate. Muscle biopsy evidenced presence of ragged red fibers (20%) and reduced COX reactivity. Assay of the muscle enzymatic activities showed multiple deficiencies of the electron transport chain and reduced ATP production. The mt.8393C>T variant in the *MT-ATP8* gene was found in homoplasmy. The patient considerably improved after valproate withdrawal.

**Conclusion:** The variant we found has been reported both as a polymorphism and, in a single patient, as related to the valproate-induced encephalopathy. The present case is the first bearing this mutation in homoplasmy. In case of neurological symptoms after starting VPA therapy, once hyperammonemia and liver failure have been ruled out, mtDNA abnormalities should be considered.

Valproic acid (VPA) is normally considered a safe and well tolerated drug, especially in adulthood; nevertheless there are several reports of patients developing both motor and cognitive neurological impairment while on therapy with VPA. Here we describe a 65-year-old woman who developed a subacute encephalopathy after VPA intake at therapeutic doses. The patient harbored a mitochondrial DNA variant, previously described as causing VPA sensitivity in one child (1).

## Case report

**Figure 1:**
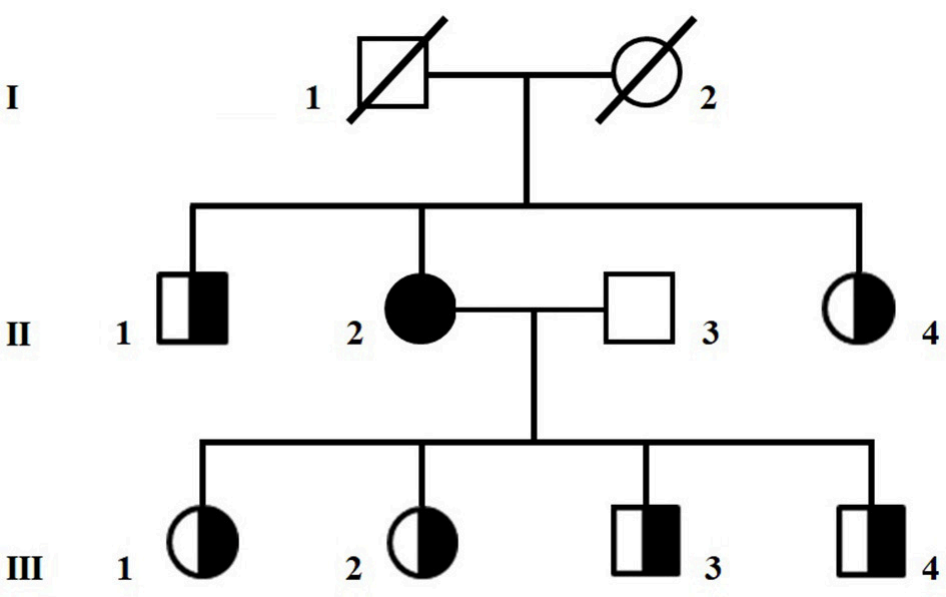
Pedigree of the family. Circles are women and squares are men. A shaded symbol indicates the proposita, half-shaded symbols indicate asymptomatic carriers of the homoplasmic m.8393C>T/p.Pro10Ser variant in *MT-ATP8*.

A 65-year old Caucasian woman with mild diabetes since 4 years, migraine and family history negative for neuromuscular diseases (Figure 1), had a few episodes of loss of consciousness and fall, sometimes associated with urinary incontinence. A CT scan showed cerebral atrophy and an EEG slowing in the theta frequency range and left temporal spike activity. Based on the suspicion that those episodes could be epileptic, she was given a daily dose of 800 mg VPA. Within 3 months of treatment, the patient had a dramatic worsening of her clinical status, becoming bedridden and lethargic.

**Figure 2:**
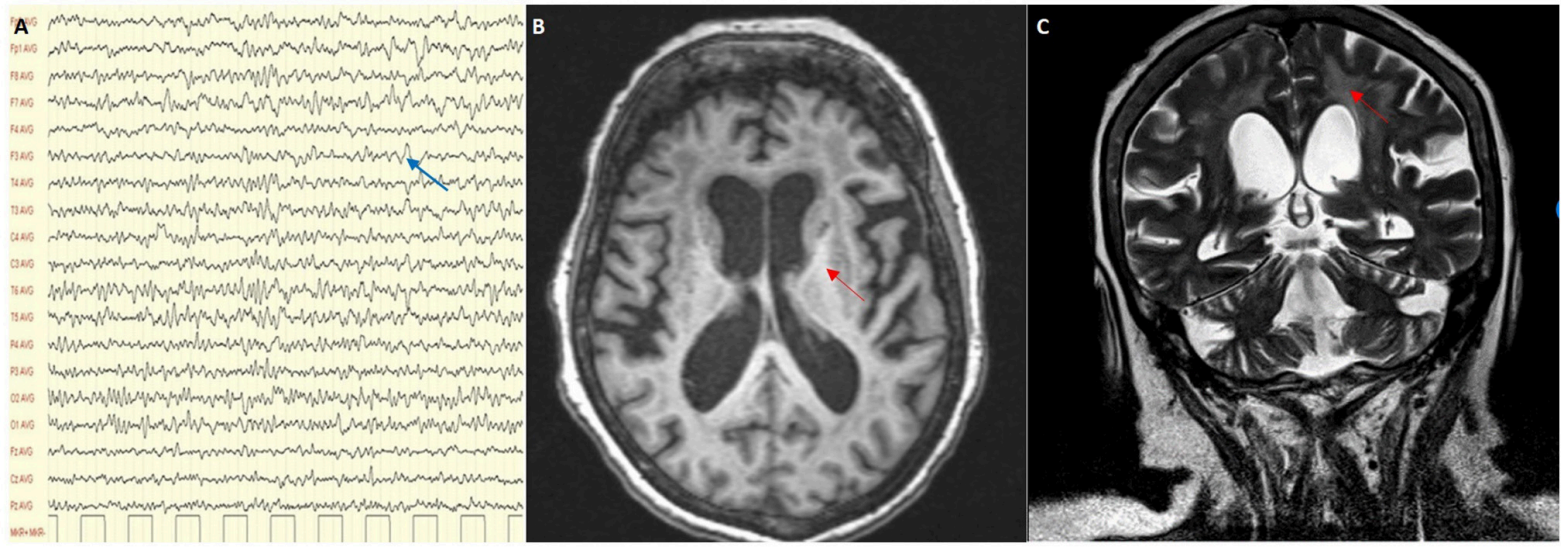
**(A)** EEG showing a slow alpha with theta and delta activity (blue arrow); **(B,C)** MRI scan showing cortical and subcortical atrophy with confluent areas of white matter hyperintensity (red arrows) in T1 and T2-weighted images.

On admission clinical examination showed a short and stocky woman (height 138 cm, weight 70 kg) who appeared drowsy, but yet oriented in space and time. She could not walk nor stand unaided. Neurological examination showed vertical gaze palsy, intact function of the other cranial nerves, including normal fundus oculi, diffuse lower limb weakness (MRC score 3), and brisk tendon reflexes, without sensory, extrapyramidal and cerebellar involvement. Blood VPA was 61.9 mcg/ml (recommended therapeutic range 50–100), blood ammonia 45 μmol/l (n.v. 11–35), and serum lactate level at rest 8.6 mmol/l (n.v. < 2.2). There was also presence of organic aciduria with intermediates of Krebs cycle in the urine. Abdomen ultrasound imaging showed moderate fatty liver and no other relevant findings. The EEG showed slow activity in the lower alpha range (7.5–9/sec), mixed to theta and delta activity over the left fronto-temporal and occipital regions (Figure 2A). Brain MRI with MR-spectroscopy (MRS) showed diffuse cortico–subcortical atrophy, more marked in the fronto-temporal region. There were large confluent areas of white matter (WM) hyperintensity in T1 and T2-weighted images at level of the semioval centers and the periventricular region bilaterally, with extension in the capsula externa and sparing of the juxtacortical “U” fibers (Figures 2B,C). MRS showed a marked reduction of the NAA spectrum in the basal ganglia and in the WM, with a double spike at 1.3 ppm compatible with a peak of lactate (not shown).

**Figure 3:**
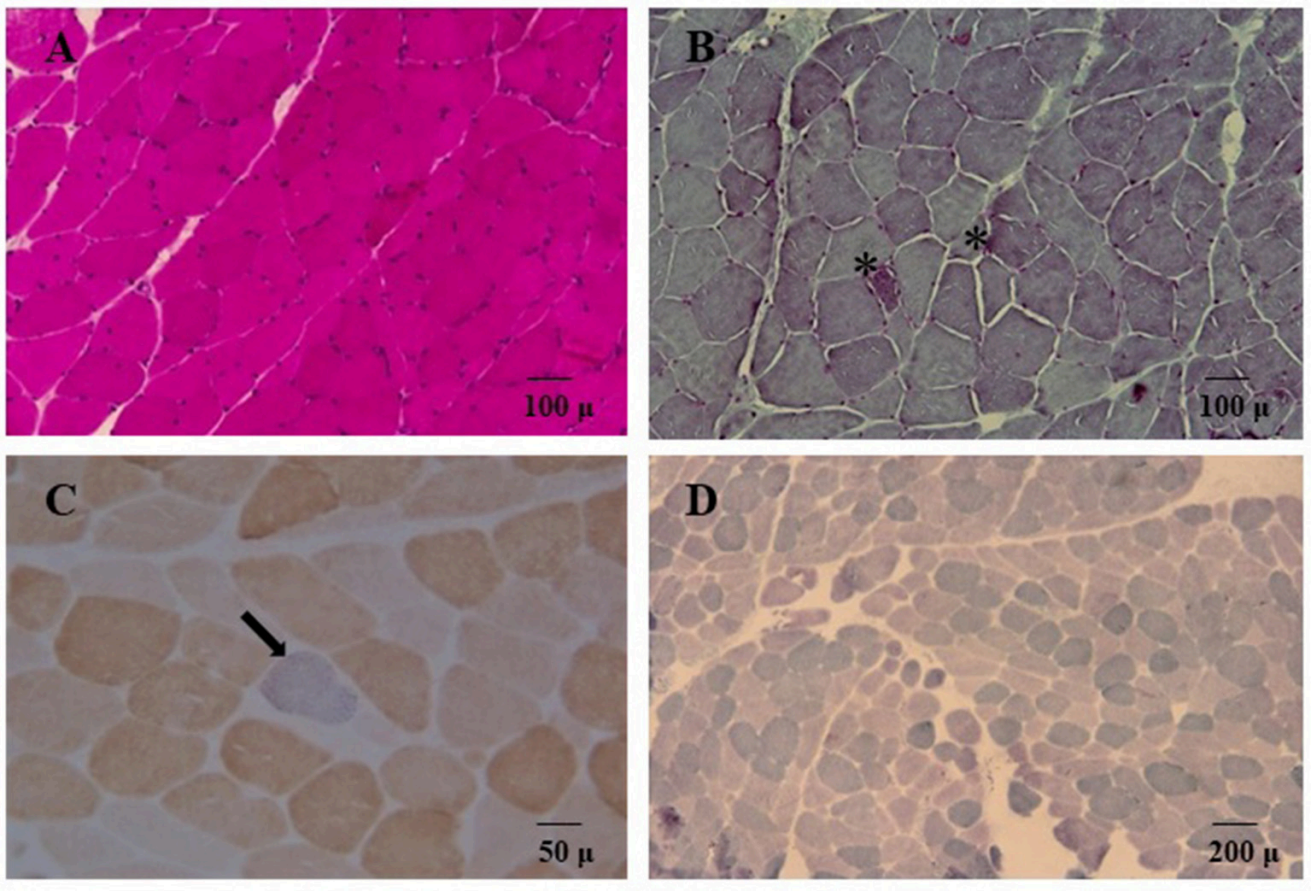
Hematoxylin and eosin stain **(A)** and Gömöri trichrome stain **(B**, 20X) showed variability of muscle morphology with some atrophic fibers. In **(B)** asterisks denote some ragged-red fibers. Double cytochrome c-oxidase/succinate dehydrogenase (COX/SDH) stain (40X) is depicted in panel **(C)**; a single COX-deficient blue fiber is indicated (arrow). In **(D)** SDH staining (10X) showed globally reduced activity.

We decided to titrate and then dismiss VPA therapy. During the following 2 weeks the patient had a significant improvement: she appeared alert and oriented, could walk unaided and came back able to perform daily activities independently. Neurological examination showed significant improvement of the lower limbs muscle strength (MRC score 4++). At a 12 month follow-up her clinical condition appeared unchanged. She underwent a muscle biopsy that evidenced some nonspecific age-related alterations as one necrotic fiber and a variability of morphology with some hypotrophic and angulated fibers and some evidence of mitochondrial dysfunction. In particular, 20% of the fibers were ragged-red at the Gomori trichrome stain with few cytochrome *c* oxidase negative (COX) fibers and slightly reduced activity of succinate dehydrogenase (SDH) (Figure 3). The contemporary alteration of COX and SDH activity at histochemistry suggested an oxidative metabolism dysfunction. Subsequently, the spectrophotometric determination of respiratory chain enzyme activities in muscle homogenate showed multiple complex enzyme deficiencies. In particular, the activities of NADH-cytochrome *c* oxidoreductate (complexes I+III), succinate cytochrome *c* oxidoreductase (complexes II+III) and COX (complex IV) were 27, 20, and 23% of normal control values, respectively, when enzyme complexes were corrected for the activity of citrate synthase, a mitochondrial matrix enzyme used as an index of total mitochondrial mass (Table 1).

**Table 1:** Respiratory chain enzyme activities in muscle homogenate.

	**Patient**	**Control values**
CI (NADH dehydrogenase)	1.73	1.56–2.60
CII (succinate:malonate dehydrogenase)	0.05	0.07–0.11
CI+CIII (NADH cytochrome *c* oxidoreductase)	0.03 (27.3%)	0.11–0.25
CII+CIII (succinate cytochrome *c* oxidoreductase)	0.01 (20.0%)	0.05–0.08
CIV (cytochrome *c* oxidase)	0.04 (23.5%)	0.17–0.28
CS (citrate synthase)	10.68	7.80–10.90

*The enzymatic activities of the respiratory chain complexes are expressed as mmol/min/gr protein and normalized to the activity of citrate synthase, a marker of mitochondrial mass. The percentage of residual activity of normal controls is shown in brackets*.

Sequencing of the whole mitochondrial DNA in peripheral blood and skeletal muscle assigned the patient to mitochondrial haplogroup X2b and detected the homoplasmic m.8393C>T/p.Pro10Ser variant in *MT-ATP8*. Large scale mitochondrial deletions were appropriately ruled out. The p.Pro10Ser variant, of uncertain pathogenic significance (www.mitomap.org/foswiki/bin/view/Main/SearchAllele) but already associated with VPA-induced toxic encephalopathy (1), was also homoplasmic in blood from six healthy relatives (Figure 1). Western blot analyses in muscle homogenate from the patient did not reveal any reduction in the expression of respiratory chain complex subunits compared to controls (Supplementary Figure 1) whereas ATP levels determined in cultured skin fibroblasts (2) showed reduced mitochondrial ATP synthesis and increased glycolytic ATP in the proposita, but not in her healthy daughter (III-02), compared to controls (Supplementary Figure 2), suggesting an impaired ATP production machinery. To investigate if additional variants in nuclear DNA-encoded mitochondrial gene could contribute to the clinical phenotype, we analyzed in blood DNA from the proposita the coding regions of 1172 nuclear genes known to be associated with mitochondrial disorders (Mito-chip, see Supplementary Information) but we failed to detect rare predictably pathogenic variants of clinical significance. Supplementary Table 1 lists rare (MAF < 0.01) variants of uncertain significance identified in the patient.

## Discussion

VPA is a medication commonly prescribed for many types of seizures, but also administered in cases of bipolar disorder and as prophylaxis of migraine headache. Its mechanism of action, though not fully understood, involves an increase in the brain concentration of gamma-aminobutyric acid (GABA), and a direct effect on the potassium channels of the neuronal membrane. Although VPA is usually well tolerated, serious complications may occur and several cases of VPA-induced encephalopathy have been reported (3–5). The features of these cases vary greatly and no definitive classification or definite pathophysiological mechanisms are available. VPA-induced encephalopathy has been described in hyperammonemic patients with or without liver failure, in patients with liver failure and normal ammonia levels, and finally in patients showing neither hepatopathy nor hyperammonemia. Our patient showed normal liver function tests and minimally increased levels of ammonia. Her EEG and MRI patterns were compatible with a toxic-metabolic encephalopathy. EEG showed diffuse slowing and predominance of theta and delta activity, as frequently reported in VPA-induced encephalopathy. Triphasic waves, and occasional bursts of frontal intermittent rhythmic delta activity (FIRDA), described in other cases (6), were not present. Her brain MRI displayed cortical and subcortical atrophy with white matter hyperintensities. Cerebral cortex and basal ganglia abnormalities, found in some patients (7, 8), were not evident. Reversible cerebral atrophy has been sometimes described (1, 9), but in our case sequential MRI studies are not available. The present case had several features suggestive of mitochondrial dysfunction (short stature, increased serum lactate, organic aciduria, MRS, and muscle biopsy findings) which prompted a study of mitochondrial biochemistry and mtDNA analysis.

VPA-induced encephalopathy can be a potentially life-threatening complication, and its early diagnosis is very important because patients can fully recover after drug discontinuation. Some experimental and clinical data suggest that early intravenous supplementation with l-carnitine could improve survival. As it does not appear to be harmful, l-carnitine is commonly recommended in severe VPA toxicity (10).

The human ATPase 8 is one of the subunits of the mitochondrial ATP synthase complex and this enzyme is responsible for most of the ATP production in cells (11). Several mutations affecting *MT-ATP8* have already been described in patients presenting with heterogeneous clinical features, varying from neurological to cardiac disorders (12). Our patient harbored a variant in *MT-ATP8*, which is likely to cause a deficit of ATP production. Yet, this mtDNA variant alone appeared not sufficient to produce clinical manifestations, as similar load of mutant mitochondrial genomes, at least in blood, was seen in six relatives of the proposita (Figure 1) and in one of them no deficit of ATP synthesis was detected (Supplementary Figure 2). Therefore, it is possible to hypothesize that VPA exposure might unmask the deleterious impact of this variant on ATP production. Silent mutations or even polymorphisms can become overt once the mitochondrial metabolism is stressed, such as during VPA therapy. Indeed, the drug has a primary mitochondrial toxic effect, through inhibition of complexes I and IV of the respiratory chain, ATP synthesis, and β-oxidation (13, 14). There are already few reports of mitochondrial syndromes triggered by VPA intake (15, 16). Other factors such as aging, the number of mtDNA copies within the cells (17), or combination of rare heterozygous variants in further nuclear mitochondrial genes (others than those tested in this study) (2) might also influence the phenotype, but this remains speculative. Further studies are needed to better define the role of the m.8393 C>T variant in *MT-ATP8* in the pathogenesis of VPA-induced encephalopathy.

The present case contributes to increase awareness of clinical neurologists toward possible mitochondrial disease when VPA-induced encephalopathy is observed. Screening of mitochondrial DNA in patients exposed to VPA and presenting an otherwise unexplained clinical worsening may be considered in cases with clinical, biochemical, or morphological features consistent with a mitochondrial dysfunction.

## Ethics statement

Written informed consent was obtained from the participants for the participation in the study and publication of this case report.

## Author contributions

GioDM and PS acquired the clinical data, drafted the manuscript and reviewed the literature. CN and AR performed genetic testing and biochemical analysis of muscle tissue. LR acquired the clinical data and performed muscle biopsy. SP and AA acquired the clinical data and reviewed the manuscript. MQ reviewed and discussed MRI findings. AF and GiuDM supervised the initial draft and critically revised the manuscript. FS oversaw data acquisition, supervised the initial draft and critically revised the manuscript.

### Conflict of interest statement

The authors declare that the research was conducted in the absence of any commercial or financial relationships that could be construed as a potential conflict of interest.

## Supplementary material

Supplementary Figure 1 A representative immunoblot analysis of muscle homogenate samples from the proposita (Pt) and a normal control (Ctrl) using specific antibodies against subunits of respiratory chain enzyme complexes CI (NDUFS7), CII (SDHB), CIII (Core2), CIV (MTCO2), and CV (ATPase-a). GAPDH was used to control for equal loading. All monoclonal antibodies are from Abcam-Mitosciences. Quantitative analysis was performed by ImageJ software (rsbweb.nih.gov/ij/). No significant differences were observed between Pt and Ctrl.

Supplementary Figure 2 Luminometric determination of glycolytic and mitochondrial adenosine triphosphate (ATP) production in skin fibroblasts from controls (Ctrls; *n* = 10), the proposita (II-2) and her daughter (III-2). Cells were incubated 2 h in ATP record buffer supplemented with either 10 mM glucose, glucose plus 2.5 mg/mL oligomycin (glycolytic ATP generation), or 5 mM 2-deoxy-D-glucose plus 5 mM pyruvate (mitochondrial ATP production). Data represent the mean ± standard deviation of three different determinations, and are expressed as percentage of the total cellular ATP production.

Supplementary Table 1 List of variants of uncertain significance with MAF < 0.01 detected in the proband using Mito-chip.
