# Supplementary figures and images for: Reversible Valproate-Induced Subacute Encephalopathy Associated With a MT-ATP8 Variant in the Mitochondrial Genome
Source: Front Neurol. 2018 Aug 30;9:728. doi: 10.3389/fneur.2018.00728 (PMC6125373; doi:10.3389/fneur.2018.00728)

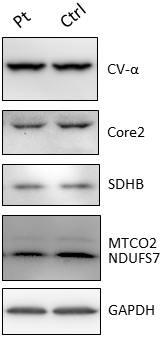

Supplement: Supplementary Figure 1 — A representative immunoblot analysis of muscle homogenate samples from the proposita (Pt) and a normal control (Ctrl) using specific antibodies against subunits of respiratory chain enzyme complexes CI (NDUFS7), CII (SDHB), CIII (Core2), CIV (MTCO2), and CV (ATPase-a). GAPDH was used to control for equal loading. All monoclonal antibodies are from Abcam-Mitosciences. Quantitative analysis was performed by ImageJ software (rsbweb.nih.gov/ij/). No significant differences were observed between Pt and Ctrl. [file Image_1.TIF]

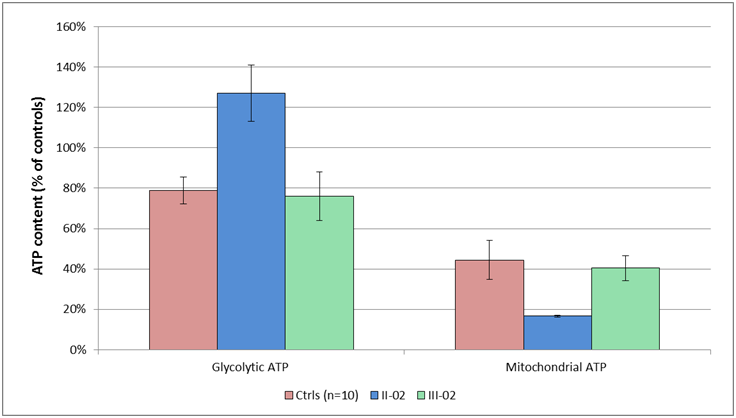

Supplement: Supplementary Figure 2 — Luminometric determination of glycolytic and mitochondrial adenosine triphosphate (ATP) production in skin fibroblasts from controls (Ctrls; n = 10), the proposita (II-2) and her daughter (III-2). Cells were incubated 2 h in ATP record buffer supplemented with either 10 mM glucose, glucose plus 2.5 mg/mL oligomycin (glycolytic ATP generation), or 5 mM 2-deoxy-D-glucose plus 5 mM pyruvate (mitochondrial ATP production). Data represent the mean ± standard deviation of three different determinations, and are expressed as percentage of the total cellular ATP production. [file Image_2.TIF]
