# Supplementary material for: Reversible Valproate-Induced Subacute Encephalopathy Associated With a MT-ATP8 Variant in the Mitochondrial Genome
Source: Front Neurol. 2018 Aug 30;9:728. doi: 10.3389/fneur.2018.00728 (PMC6125373; doi:10.3389/fneur.2018.00728)
Supplement: Supplementary Table 1 — List of variants of uncertain significance with MAF < 0.01 detected in the proband using Mito-chip. [file Table_1.DOCX]

| **Gene Symbol** | **Variant** |
| --- | --- |
| *ACAD10* | c.1330A>C/p.K444Q |
| *CCT7* | c.1356T>A/p.N452K |
| *COQ8B* | c.1493C>A/p.A498D |
| *CPS1* | c.972T>G/p.N324K |
| *FAM210B* | c.373A>G/p.M125V |
| *FDXR* | c.1588C>T/p.R530C |
| *GRSF1* | c.958G>A/p.E320K |
| *MIPEP* | c.1476T>G/p.N492K |
| *RHOT2* | c.1001G>T/p.S334I |
| *SLC37A4* | c.628T>C/p.S210P |
| *TOP1MT* | c.193C>A/p.P65T |

**Supplementary Table** List of variants of uncertain significance with MAF < 0.01 detected in the proband using Mito-chip.
