# Supplementary material for: Reversible Valproate-Induced Subacute Encephalopathy Associated With a MT-ATP8 Variant in the Mitochondrial Genome
Source: Front Neurol. 2018 Aug 30;9:728. doi: 10.3389/fneur.2018.00728 (PMC6125373; doi:10.3389/fneur.2018.00728)
Supplement: Supplementary file 4 [file Table_2.DOC]

**SUPPORTING INFORMATION**

We designed an extended next-generation sequencing (NGS) testing panel designed with the NimbleGen Design software for the SeqCap EZ target enrichment system (Roche, Madison, WI, USA), adapted for a MiSeq desktop sequencer (Illumina, San Diego, CA, USA).

This multigene panel, termed *MitoChip,* targeted the coding exons and 50-bp of the flanking introns of 1172 genes with mitochondrial targeting all enlisted in MitoCarta 2.0 (1), as well as additional genes selected after a careful literature search.

*MitoChip* comprises the following genes: *AADAT, AARS2, AASS, ABAT, ABCA13, ABCA9, ABCB10, ABCB6, ABCB7, ABCB8, ABCB9, ABCD1, ABCD2, ABCD3, ABCF2, ABHD10, ABHD11, ACAA1, ACAA2, ACACA, ACACB, ACAD10, ACAD11, ACAD8, ACAD9, ACADL, ACADM, ACADS, ACADSB, ACADVL, ACAT1, ACCS, ACLY, ACO1, ACO2, ACOT13, ACOT2, ACOT7, ACOT9, ACOX1, ACOX3, ACP6, ACSF2, ACSF3, ACSL1, ACSL4, ACSL6, ACSM1, ACSM2A, ACSM3, ACSM5, ACSS1, ACSS3, ACYP2, ADCK1, ADCK2, ADCK3, ADCK4, ADCK5, ADHFE1, AFG3L2, AGK, AGMAT, AGPAT5, AGR2, AGXT, AGXT2, AHCYL1, AIFM1, AIFM2, AIFM3, AK2, AK3, AK4, AKAP1, AKAP10, AKR1B10, AKR7A2, ALAS1, ALAS2, ALDH18A1, ALDH1B1, ALDH1L1, ALDH1L2, ALDH2, ALDH3A2, ALDH4A1, ALDH5A1, ALDH6A1, ALDH7A1, ALDH9A1, ALKBH1, ALKBH3, ALKBH7, AMACR, AMT, ANGEL2, APEX2, APOA1BP, APOO, APOOL, APOPT1, APTX, ARF5, ARG2, ARL2, ARMC10, ARMS2, ASAH2, ATAD1, ATAD3A, ATAD3B, ATIC, ATP10D, ATP5A1, ATP5B, ATP5C1, ATP5D, ATP5E, ATP5F1, ATP5G1, ATP5G2, ATP5G3, ATP5H, ATP5I, ATP5J, ATP5J2, ATP5J2-PTCD1, ATP5L, ATP5O, ATP5S, ATP5SL, ATPAF1, ATPAF2, ATPIF1, ATXN2, AUH, AURKAIP1, BAD, BAK1, BAX, BCAT2, BCKDHA, BCKDHB, BCKDK, BCL2, BCL2L1, BCL2L13, BCL2L2, BCS1L, BDH1, BID, BLOC1S1, BNIP3, BNIP3L, BOLA1, BOLA3, BPHL, C10orf10, C10orf2, C12orf10, C12orf65, C14orf159, C14orf2, C15orf40, C15orf48, C15orf61, C16orf91, C17orf89, C19orf12, C19orf52, C19orf70, C1QBP, C20orf24, C21orf33, C2orf47, C2orf69, C3orf33, C5orf63, C6orf136, C6orf203, C7orf55, C8orf82, CA5A, CA5B, CARKD, CARS2, CASP8, CAT, CBR3, CBR4, CCBL2, CCDC109B, CCDC127, CCDC51, CCDC58, CCDC90B, CCT7, CDC25C, CECR5, CEP89, CHCHD1, CHCHD10, CHCHD2, CHCHD3, CHCHD4, CHCHD5, CHCHD6, CHCHD7, CHDH, CHPT1, CISD1, CISD2, CISD3, CKMT1A, CKMT1B, CKMT2, CLIC4, CLPB, CLPP, CLPX, CLYBL, CMC1, CMC2, CMC4, CMPK2, COA1, COA3, COA4, COA5, COA6, COA7, COASY, COMT, COMTD1, COQ10A, COQ10B, COQ2, COQ3, COQ4, COQ5, COQ6, COQ7, COQ9, COX10, COX11, COX14, COX15, COX16, COX17, COX18, COX19, COX20, COX4I1, COX4I2, COX5A, COX5B, COX6A1, COX6A2, COX6B1, COX6B2, COX6C, COX7A1, COX7A2, COX7A2L, COX7B, COX7C, COX8A, COX8C, CPOX, CPS1, CPT1A, CPT1B, CPT1C, CPT2, CRAT, CRLS1, CROT, CRY1, CRYZ, CS, CYB5A, CYB5B, CYB5R2, CYB5R3, CYC1, CYCS, CYP11A1, CYP11B1, CYP11B2, CYP24A1, CYP27A1, CYP27B1, D2HGDH, DAP3, DARS, DARS2, DBI, DBT, DCAKD, DCXR, DDAH1, DDHD1, DDX28, DECR1, DGUOK, DHCR24, DHODH, DHRS1, DHRS4, DHRS7B, DHRSX, DHTKD1, DHX30, DIABLO, DLAT, DLD, DLST, DMGDH, DMPK, DNA2, DNAJA3, DNAJC11, DNAJC15, DNAJC19, DNAJC28, DNAJC30, DNAJC4, DNLZ, DNM1L, DPYD, DTYMK, DUS2, DUSP26, DUT, EARS2, ECH1, ECHDC1, ECHDC2, ECHDC3, ECHS1, ECI1, ECI2, ECSIT, EEFSEC, EFHD1, EHHADH, ELAC2, EMC2, ENDOG, EPHX2, ERAL1, ETFA, ETFB, ETFDH, ETHE1, EXOG, FABP1, FAHD1, FAHD2A, FAM136A, FAM162A, FAM185A, FAM195A, FAM210A, FAM210B, FAM213A, FARS2, FASN, FASTK, FASTKD1, FASTKD2, FASTKD3, FASTKD5, FBXL4, FDPS, FDX1, FDX1L, FDXR, FECH, FH, FHIT, FIS1, FKBP10, FKBP8, FLAD1, FOXRED1, FPGS, FTH1, FTMT, FTSJ2, FUNDC1, FUNDC2, FXN, GADD45GIP1, GAPDH, GARS, GATB, GATC, GATM, GBAS, GCAT, GCDH, GCSH, GDAP1, GFER, GFM1, GFM2, GHITM, GK, GLDC, GLOD4, GLRX2, GLRX5, GLS, GLS2, GLUD1, GLYAT, GLYCTK, GNG5, GOLPH3, GOT2, GPAM, GPD2, GPI, GPT2, GPX1, GPX4, GRHPR, GRPEL1, GRPEL2, GRSF1, GSR, GSTK1, GSTO1, GSTZ1, GTPBP10, GTPBP3, GTPBP6, GUF1, GUK1, HADH, HADHA, HADHB, HAGH, HAO2, HARS2, HCCS, HDHD3, HEBP1, HEMK1, HIBADH, HIBCH, HIGD1A, HIGD2A, HINT1, HINT2, HINT3, HK1, HK2, HLCS, HMBS, HMGCL, HMGCS2, HOGA1, HRSP12, HSCB, HSD17B10, HSD17B4, HSD17B8, HSDL1, HSDL2, HSPA9, HSPB7, HSPD1, HSPE1, HTATIP2, HTRA2, IARS2, IBA57, ICT1, IDE, IDH1, IDH2, IDH3A, IDH3B, IDH3G, IDI1, IFI27, IMMP1L, IMMP2L, IMMT, ISCA1, ISCA2, ISCU, ISOC2, IVD, KARS, KIAA0100, KIAA0141, KIAA0391, KIF1B, KMO, KRT5, L2HGDH, LACE1, LACTB, LACTB2, LAMC1, LAP3, LARS, LARS2, LDHAL6B, LDHB, LDHD, LETM1, LETM2, LETMD1, LIAS, LIPT1, LIPT2, LONP1, LONP2, LRPPRC, LRRK2, LYPLA1, LYPLAL1, LYRM1, LYRM2, LYRM4, LYRM5, LYRM7, LYRM9, MACROD1, MALSU1, MAOA, MAOB, MARC1, MARC2, MARCH5, MARS2, MAVS, MCAT, MCCC1, MCCC2, MCEE, MCU, MCUR1, MDH1, MDH2, ME1, ME2, ME3, MECR, METAP1D, METTL15, METTL17, METTL5, METTL8, MFF, MFN1, MFN2, MGARP, MGME1, MGST1, MGST3, MICU1, MICU2, MIEF1, MINOS1, MIPEP, MLH1, MLYCD, MMAA, MMAB, MMACHC, MMADHC, MOCS1, MPC1, MPC2, MPST, MPV17, MPV17L, MPV17L2, MRM1, MRPL1, MRPL10, MRPL11, MRPL12, MRPL13, MRPL14, MRPL15, MRPL16, MRPL17, MRPL18, MRPL19, MRPL2, MRPL20, MRPL21, MRPL22, MRPL23, MRPL24, MRPL27, MRPL28, MRPL3, MRPL30, MRPL33, MRPL34, MRPL35, MRPL36, MRPL37, MRPL38, MRPL39, MRPL4, MRPL40, MRPL41, MRPL42, MRPL43, MRPL44, MRPL45, MRPL46, MRPL47, MRPL48, MRPL49, MRPL50, MRPL51, MRPL52, MRPL53, MRPL54, MRPL55, MRPL57, MRPL9, MRPS10, MRPS11, MRPS12, MRPS14, MRPS15, MRPS16, MRPS17, MRPS18A, MRPS18B, MRPS18C, MRPS2, MRPS21, MRPS22, MRPS23, MRPS24, MRPS25, MRPS26, MRPS27, MRPS28, MRPS30, MRPS31, MRPS33, MRPS34, MRPS35, MRPS36, MRPS5, MRPS6, MRPS7, MRPS9, MRRF, MRS2, MSRA, MSRB2, MSRB3, MTCH1, MTCH2, MTCP1, MTERF1, MTERF2, MTERF3, MTERF4, MTFMT, MTFP1, MTFR1, MTFR1L, MTG1, MTG2, MTHFD1, MTHFD1L, MTHFD2, MTHFD2L, MTHFS, MTIF2, MTIF3, MTO1, MTPAP, MTRF1, MTRF1L, MTX1, MTX2, MUL1, MUT, MUTYH, NADK2, NAGS, NARS2, NBR1, NCEH1, NCOA4, NDUFA1, NDUFA10, NDUFA11, NDUFA12, NDUFA13, NDUFA2, NDUFA3, NDUFA4, NDUFA5, NDUFA6, NDUFA7, NDUFA8, NDUFA9, NDUFAB1, NDUFAF1, NDUFAF2, NDUFAF3, NDUFAF4, NDUFAF5, NDUFAF6, NDUFAF7, NDUFB1, NDUFB10, NDUFB11, NDUFB2, NDUFB3, NDUFB4, NDUFB5, NDUFB6, NDUFB7, NDUFB8, NDUFB9, NDUFC1, NDUFC2, NDUFS1, NDUFS2, NDUFS3, NDUFS4, NDUFS5, NDUFS6, NDUFS7, NDUFS8, NDUFV1, NDUFV2, NDUFV3, NEU4, NFS1, NFU1, NGRN, NIF3L1, NIPSNAP1, NIPSNAP3A, NIPSNAP3B, NIT1, NIT2, NLN, NLRX1, NME1, NME3, NME4, NME6, NMNAT3, NNT, NOA1, NRD1, NSUN3, NSUN4, NT5C, NT5DC2, NT5DC3, NT5M, NTHL1, NUBPL, NUCB2, NUDT13, NUDT19, NUDT2, NUDT5, NUDT6, NUDT8, NUDT9, NUP62, OAT, OBSCN, OCIAD1, OCIAD2, OGDH, OGDHL, OGG1, OMA1, OPA1, OPA3, OSBPL1A, OSGEPL1, OTC, OXA1L, OXCT1, OXLD1, OXNAD1, OXR1, OXSM, P4HB, PABPC5, PACSIN2, PAICS, PAK7, PAM16, PANK2, PARK2, PARK7, PARL, PARS2, PC, PCBD2, PCCA, PCCB, PCK2, PDC, PDE12, PDF, PDHA1, PDHA2, PDHB, PDHX, PDK1, PDK2, PDK3, PDK4, PDP1, PDP2, PDPR, PDSS1, PDSS2, PET100, PET117, PEX11B, PGAM5, PGS1, PHB, PHB2, PHYH, PI4KA, PICK1, PIF1, PINK1, PISD, PITRM1, PKLR, PLGRKT, PMAIP1, PMPCA, PMPCB, PNPLA8, PNPO, PNPT1, POLDIP2, POLG, POLG2, POLRMT, PPA2, PPIF, PPM1K, PPOX, PPTC7, PPWD1, PRDX2, PRDX3, PRDX4, PRDX5, PRDX6, PRELID1, PRELID2, PREPL, PRODH, PRODH2, PROSC, PRSS35, PSMA6, PSTK, PTCD1, PTCD2, PTCD3, PTGES2, PTPMT1, PTPN4, PTRH1, PTRH2, PTS, PUS1, PUSL1, PXMP2, PXMP4, PYCR1, PYCR2, PYURF, QDPR, QRSL1, QTRT1, RAB11FIP5, RAB24, RAB32, RAB35, RARS, RARS2, RBFA, RCN2, RDH11, RDH13, RDH14, RECQL4, REXO2, RFK, RHOT1, RHOT2, RMDN1, RMDN3, RMND1, RNASEH1, RNASEH2B, RNMTL1, ROMO1, RPIA, RPL10A, RPL34, RPL35A, RPS14, RPS15A, RPS18, RPUSD3, RPUSD4, RRM2B, RSAD1, RTN4IP1, SACS, SAMM50, SARDH, SARS2, SCCPDH, SCO1, SCO2, SCP2, SDHA, SDHAF1, SDHAF2, SDHAF3, SDHAF4, SDHB, SDHC, SDHD, SDR39U1, SDSL, SECISBP2, SEPT4, SERAC1, SERHL2, SETD9, SFXN1, SFXN2, SFXN3, SFXN4, SFXN5, SHMT1, SHMT2, SIRT3, SIRT4, SIRT5, SLC16A1, SLC16A11, SLC16A2, SLC16A7, SLC19A2, SLC19A3, SLC22A4, SLC25A1, SLC25A10, SLC25A11, SLC25A12, SLC25A13, SLC25A14, SLC25A15, SLC25A16, SLC25A17, SLC25A18, SLC25A19, SLC25A20, SLC25A21, SLC25A22, SLC25A23, SLC25A24, SLC25A25, SLC25A26, SLC25A27, SLC25A28, SLC25A29, SLC25A3, SLC25A30, SLC25A31, SLC25A32, SLC25A33, SLC25A34, SLC25A35, SLC25A36, SLC25A37, SLC25A38, SLC25A39, SLC25A4, SLC25A40, SLC25A41, SLC25A42, SLC25A43, SLC25A44, SLC25A45, SLC25A46, SLC25A47, SLC25A48, SLC25A5, SLC25A51, SLC25A53, SLC25A6, SLC30A6, SLC30A9, SLC37A4, SLC52A1, SLC52A2, SLC52A3, SLIRP, SLMO1, SLMO2, SMDT1, SNAP29, SND1, SOD1, SOD2, SPATA19, SPATA20, SPG7, SPR, SPRYD4, SPTLC2, SQRDL, SSBP1, STAR, STARD7, STOM, STOML1, STOML2, STX17, STXBP1, SUCLA2, SUCLG1, SUCLG2, SUGCT, SUOX, SUPV3L1, SURF1, SYNJ2BP, TACO1, TAMM41, TANGO2, TARS, TARS2, TAZ, TBRG4, TCAIM, TCHP, TCIRG1, TDRKH, TEFM, TFAM, TFB1M, TFB2M, THEM4, THG1L, THNSL1, TIMM10, TIMM10B, TIMM13, TIMM17A, TIMM17B, TIMM21, TIMM22, TIMM23, TIMM44, TIMM50, TIMM8A, TIMM8B, TIMM9, TIMMDC1, TK2, TKT, TMBIM4, TMEM11, TMEM126A, TMEM126B, TMEM143, TMEM14C, TMEM177, TMEM186, TMEM205, TMEM65, TMEM70, TMLHE, TOMM20, TOMM22, TOMM34, TOMM40, TOMM40L, TOMM5, TOMM6, TOMM7, TOMM70A, TOP1MT, TOP3A, TPI1, TRAP1, TRIAP1, TRIT1, TRMT1, TRMT10C, TRMT11, TRMT2B, TRMT5, TRMT61B, TRMU, TRNT1, TRUB2, TSFM, TSPO, TST, TSTD1, TTC19, TUBB3, TUFM, TXN2, TXNDC12, TXNRD1, TXNRD2, TYMP, TYSND1, UCP1, UCP2, UCP3, UNG, UQCC1, UQCC2, UQCC3, UQCR10, UQCR11, UQCRB, UQCRC1, UQCRC2, UQCRFS1, UQCRH, UQCRQ, USMG5, VARS2, VDAC1, VDAC2, VDAC3, VPS13C, VWA8, WARS2, WBSCR16, WDR81, WFS1, XPNPEP3, XRCC6BP1, YARS2, YBEY, YME1L1, ZADH2.*

Target enrichment and amplification were performed following the SeqCap EZ HyperCap Library protocol;(Roche, Madison, WI, USA), purification using Agencourt AMPure XP beads (Beckman Coulter, Inc., CA), and sequencing on a MiSeq desktop scanner according to described methodologies (2). Alignment and variant calling used the following criteria: coverage of >95% of coding exons at least 10X (and at least 90% at 20X), mean quality score (Q score) ≥30, variant selection criteria if present in ≥ 50% of the reads indicated in Integrative Genomics Viewer software, as described elsewhere (3).

**Bioinformatics**

The analysis for biological interpretation of all the variants found was performed using the Ingenuity Variant Analysis software (http://www.ingenuity.com/products/variant-analysis). Single nucleotide variant and insertion and deletion (indel) data were filtered as follows: i) exclusion of variants outside the targeted genes; ii) exclusion of synonymous variants or changes not affecting canonical sites for splicing; iii) exclusion of some recurring false-positive calls present only in reads from one restriction enzyme fragment upon scrutiny with Integrative Genomics Viewer; iv) retention only of rare variants with MAF <0.01 in the 1000G database (http://www.1000genomes.org/ accessed 12/2015), dbSNP database version 146 (http://www.ncbi.nlm.nih.gov/SNP/ accessed 12/ 2015), ESP6500SI-V2 database (evs.gs.washington.edu/, accessed 01/2016), gnomAD browser (gnomad.broadinstitute.org, version 2.0) and ExAC 3.0 dataset (exac.broadinstitute.org, accessed 6/20168), as well as in the Sequencing Initiative Suomi (SISu) database (http://sisu.fimm.fi/, accessed 06/2018).

Novel missense variants were systematically evaluated for the functional consequences *in silico* using Polyphen2 (http://genetics.bwh.harvard.edu/pph2/), SIFT, Sorting Intolerant From Tolerant (http://sift.jcvi.org/), and Alamut (www.interactive-biosoftware.com/) predictions. To test possible deleterious effects of synonymous and missense variants on splicing we used the Human Splicing Finder web tool.

**References**

1. Calvo SE, Clauser KR, Mootha VK. MitoCarta2.0: an updated inventory of mammalian mitochondrial proteins. Nucleic Acids Res. 2016; 44(D1):D1251-7.

2. Ylikallio E, Johari M, Konovalova S, et al. Targeted next-generation sequencing reveals further genetic heterogeneity in axonal Charcot-Marie-Tooth neuropathy and a mutation in HSPB1. Eur. J. Hum. Genet. 2014; 22: 522-527.

3. Sulonen AM, Ellonen P, Almusa H, et al. Comparison of solution-based exome capture methods for next generation sequencing. Genome Biol. 2011; 12: R94.
